# A Cross-Sectional Study Comparing Oxidative Stress in Patients with Epilepsy Treated with Old and New Generation Antiseizure Medications

**DOI:** 10.3390/medicina60081299

**Published:** 2024-08-12

**Authors:** Boštjan Martinc, Iztok Grabnar, Daniela Milosheska, Bogdan Lorber, Tomaž Vovk

**Affiliations:** 1 Faculty of Pharmacy, University of Ljubljana, 1000 Ljubljana, Slovenia; bostjan.martinc@gmail.com (B.M.); iztok.grabnar@ffa.uni-lj.si (I.G.);; 2 Department of Neurology, University Medical Center Ljubljana, 1000 Ljubljana, Slovenia; bogdan.lorber@kclj.si

**Keywords:** antiseizure medications, antioxidants, epilepsy, new generation ASMs, old generation ASMs, oxidative stress, poly-therapy with ASMs

## Abstract

*Background and Objectives:* Oxidative stress resulting from a disturbance of the endogenous redox system is suspected in numerous diseases of the central nervous system, including epilepsy. In addition, antiseizure medications (ASMs), especially those of the old generation, may further increase oxidative stress. To evaluate the effects of ASM generation on oxidative stress, we conducted a cross-sectional study in patients with epilepsy treated with old, new, and polytherapy. *Materials and Methods*: The antioxidant activity of superoxide dismutase, catalase, glutathione peroxidase, and glutathione reductase, as well as the concentrations of malondialdehyde, protein carbonyl, nitrate, nitrite, and glutathione in reduced and oxidized forms, were measured in 49 patients with epilepsy and 14 healthy controls. In addition, the plasma concentrations of ASMs and metabolites of carbamazepine and valproic acid were measured in the patients. *Results*: Patients with epilepsy showed increased activities of superoxide dismutase and catalase (*p* < 0.001), concentrations of glutathione disulfide and markers of nitric oxide metabolism (*p* < 0.001), and decreased activities of glutathione peroxidase, glutathione reductase, glutathione, and nitrite concentrations (*p* ≤ 0.005) compared to healthy controls. A comparison of ASM generations revealed increased levels of superoxide dismutase and catalase (*p* ≤ 0.007) and decreased levels of glutathione peroxidase and glutathione reductase (*p* ≤ 0.01) in patients treated with old ASMs compared to those treated with new generation ASMs. In addition, an increase in protein carbonyl and nitric oxide metabolites (*p* ≤ 0.002) was observed in patients treated with old generation ASMs compared to those treated with new generation ASMs. Most oxidative stress parameters in patients receiving polytherapy with ASMs were intermediate between the results of patients treated with the old and new generations of ASMs. *Conclusions*: An increase in oxidative stress markers and modulation of antioxidant enzyme activities was observed in patients with epilepsy compared to controls. The results of our study showed significantly higher oxidative stress in patients treated with old ASMs compared to those treated with new generation ASMs.

## 1. Introduction

Epilepsy is one of the most common and heterogeneous neurological diseases, characterized by recurring unprovoked (or reflex) epileptic seizures or a high recurrence risk for epileptic seizures. It has significant neurobiological, psychological, cognitive, and social consequences [1].

The International League Against Epilepsy (ILAE) categorizes the etiology of epilepsy into several distinct groups: structural (characterized by the presence of a lesion potentially associated with epilepsy), genetic (linked to identifiable mutations), infectious, metabolic, immune, and idiopathic origins. In accordance with the ILAE’s 2017 classification, epileptic seizures are classified into three primary categories: focal onset seizures (originating in a specific hemisphere), generalized onset seizures (initiating simultaneously in both hemispheres), and seizures of unknown onset [2]. It has been shown that the formation of reactive species or the decrease in activity of endogenous antioxidant systems may result in different forms of epilepsy as well as increased chances of repeating epileptic seizures [3].

Nowadays, the potential role of oxidative stress in central nervous system disorders is already well established [4]. The brain is more prone to oxidative damage since it has a high demand for oxygen, its antioxidant defense mechanisms are particularly poor, and it contains a large amount of easily oxidized unsaturated fatty acids. In addition, the brain is also rich in iron, which can catalyze reactions with free radicals [5].

Under normal physiological conditions, the endogenous antioxidant defense system, which includes enzymatic and non-enzymatic antioxidants, is able to successfully control exposure to reactive species and thus protect biological macromolecules from potential oxidative damage. The primary antioxidant enzymes are represented by superoxide dismutase (SOD), catalase (CAT), glutathione peroxidase (GPx), and glutathione reductase (GR). These enzymes act together and simultaneously at different points of the metabolic pathway of free radicals. A possible failure of this antioxidant defense system can therefore lead to oxidative stress and, thus, cause damage to biological macromolecules.

To confirm this theory, the potential role of oxidative stress in epilepsy has been extensively studied in drug-naïve patients compared to healthy controls [6,7,8,9,10,11,12,13,14,15] and in patients treated with different antiseizure medications (ASMs) compared to drug-naïve patients or healthy controls [11,12,13,14,16,17,18,19,20]. Clinical studies in drug-naïve patients with epilepsy have shown that epilepsy itself can affect the endogenous antioxidative-oxidative system. Thus, the majority of published studies have confirmed increased oxidative stress in drug-naïve patients compared to healthy controls, particularly through the observed increase in markers of oxidative damage to biological macromolecules. Most clinical studies therefore show increased lipid peroxidation markers [9,11,12,13,21,22,23], protein carbonyl (PC) [11,22] and 8-hydroxy-2′-deoxyguanosine [21], the latter two serving as markers of oxidative damage to proteins and nucleic acids, respectively. On the other hand, there are only a few studies that show unchanged [7,14,24,25,26] or even decreased [27] lipid peroxidation markers. Moreover, the positive correlation between epilepsy and markers of lipid peroxidation was confirmed by a meta-analysis of the previously mentioned clinical studies investigating oxidative stress in drug-naïve patients with epilepsy [28].

Although there is an apparent correlation between epilepsy and markers of oxidative damage to biological macromolecules, it is difficult to draw a credible and definitive conclusion about the impact of epilepsy on other markers of oxidative stress, including the activities of antioxidant enzymes (SOD, CAT, GPx, and GR), nitric oxide (NO) levels, and glutathione (GSH) concentrations, as the results from a number of published clinical studies are inconsistent [9,11,13,21].

The primary approach to epilepsy treatment, which is suitable for about 70% of patients with epilepsy, is pharmacological treatment [29]. A wide variety of ASMs are generally divided into generations depending on the date of their introduction into clinical use. The main representatives of the old generation are carbamazepine (CBZ), phenobarbital (PB), phenytoin (PHT), and valproic acid (VPA), while lamotrigine (LTG), levetiracetam (LEV), oxcarbazepine (OXC), pregabalin (PGB), topiramate (TPM), vigabatrin (VGB), and lacosamide are the main drugs of the new generation [4,30]. It has been shown that treatment with ASMs can additionally influence and even modulate the endogenous oxidant/antioxidant balance [7,31]. Several clinical studies reported an increase in malondialdehyde (MDA) levels in the VPA-treated group [25,32,33], while in the CBZ group, the overall effect of treatment on MDA levels was inconclusive [12,32,34]. Moreover, these results were further confirmed in our meta-analysis, which showed a significantly increased level of oxidative stress by measuring lipid peroxidation markers in the case of treatment with VPA, while no statistically significant changes were observed in the case of treatment with CBZ [28]. In addition, some other studies with other ASMs, such as PB [7] and PHT [35,36] have similarly confirmed increased lipid peroxidation. Furthermore, free radical formation could be a possible cause of numerous side effects of ASMs and even the failure of seizure control.

However, a major shortcoming of these studies is that in the majority of the published studies that investigated the influence of various ASMs on oxidative stress in epilepsy, mainly the above-mentioned representatives of the old generation ASM, mostly CBZ, VPA, FB, PHT, or their combinations, were investigated, and only a few studies evaluated the effect of ASMs of the new generation.

Although there is limited data evaluating the effect of the new ASMs, the existing clinical studies generally show a better oxidative profile. Studies on OXC [26,37], TPM [33], and LTG [31,36] have shown lower lipid peroxidation compared to untreated patients with epilepsy or healthy controls. On the other hand, studies in patients treated with LEV showed increased 8-hydroxy-2′-deoxyguanosine concentration [17] and increased lipid peroxidation markers [38], compared to untreated controls.

As there is a lack of studies investigating the influence of new generation ASMs and the difference between old and new generation ASMs on oxidative stress markers in patients with epilepsy, we decided to conduct a cross-sectional study in patients with epilepsy treated with different ASMs.

## 2. Materials and Methods

### 2.1. Study Population and Clinical Assessments

In this cross-sectional observational study, oxidative stress parameters were investigated in patients with epilepsy and healthy controls. In addition, the effects of different generations of ASMs on oxidative stress parameters in patients with epilepsy were investigated. The study compared oxidative stress parameters in patients treated with old and new generation ASMs and with polytherapy. All patients and healthy volunteers were required to provide written informed consent prior to enrollment in the study. The study was approved by the National Medical Ethics Committee of the Republic of Slovenia (registration number: 152/06/10).

Participants underwent a comprehensive assessment, which included demographic data, duration of epilepsy, seizure frequency, presence of comorbidities, drug therapy, concomitant use of over-the-counter medications and dietary supplements, and potential systemic, neurological, and rare idiosyncratic ASM side effects. The inclusion criteria were focal or generalized epilepsy, treatment with CBZ, VPA, LEV, PGB, or TPM for at least 3 months, both sexes, age over 18 years, and provision of written informed consent.

The exclusion criteria included advanced neurodegenerative and cerebrovascular diseases, cancer, endocrinologic disorders, inflammatory diseases, thyroid, liver, kidney, and heart diseases, chronic diseases such as diabetes, hypertension, hyperlipidemia, and rheumatoid arthritis, recent surgery, use of anti-inflammatory drugs (non-steroidal anti-inflammatory drugs and corticosteroids) or antioxidants (vitamins and mineral supplements), smoking, alcohol abuse, and a BMI of over 30 kg/m^2^. Pregnant women were also not included in the study.

The included patients with epilepsy received monotherapy or polytherapy with ASMs. Based on the generation of ASMs, they were divided into the old generation, the new generation, and the polytherapy group [30,39]. Patients in the old generation ASM group received monotherapy with CBZ or VPA, while patients in the new generation group received monotherapy with LEV, PGB, or TPM. Patients treated with more than one ASM were assigned to the polytherapy group.

### 2.2. Blood Sampling and Assays

Venous blood was collected in EDTA- and heparin-coated Vacutainers and centrifuged. Whole blood, plasma, and cell samples were aliquoted into separate tubes and stored at −80 °C for further analysis.

The oxidative stress parameters analyzed were MDA, PC, NO metabolites, glutathione, and antioxidant enzymes.

Oxidative damage to biological macromolecules was assessed by measuring MDA and PC. Lipid peroxidation was estimated by measuring MDA levels using liquid chromatography with tandem mass spectrometry (LC-MS/MS). The MDA content was determined after alkaline hydrolysis of protein-bound MDA and subsequent derivatization with 2,4-dinitrophenylhydrazine to a hydrazone product in acidic medium according to a previously published method [40]. Based on the same reaction mechanism, PC concentrations were determined using the Cayman Chemical Company Protein Carbonyl Assay Kit (Ann Arbor, MI, USA). The concentrations of MDA and PC were expressed as µmol per liter of plasma.

NO metabolism was determined by measuring the total nitrate/nitrite and nitrite levels in plasma using the Cayman colorimetric nitrate/nitrite kit (Ann Arbor, MI, USA).

The concentrations of reduced (GSH) and oxidized (GSSG) forms of glutathione were determined in whole blood samples using the LC-MS/MS method according to the published method with some minor modifications [41].

The activities of endogenous antioxidant enzymes were measured in whole blood using the Ransel^®^ kit for GPx and in blood cells using the Ransod^®^ kit for SOD and Glutathione reductase kit for GR. All kits are from Randox^®^ (Crumlin, UK). Catalase (CAT) activity was determined in blood cells according to the modified method described by Aebi [42]. The activities of the antioxidant enzymes were expressed in units per ml or l of whole blood or plasma.

The concentrations of ASMs were determined in plasma samples according to previously published methods for CBZ and its main metabolite carbamazepine-10,11-epoxide [43], LEV [44], PGB [45], TPM [45], and VPA and its metabolite 2-propyl-4-pentenoic acid [46].

### 2.3. Statistical Analysis

The data are presented as medians, frequencies, or percentages. The comparison between the two groups was made using the Fischer exact test for categorical variables and the Mann—Whitney U test for continuous variables. The comparison between the three groups was carried out using the Kruskal—Wallis test and the Mann—Whitney U test with a Holm—Bonferroni correction for multiple comparisons. Spearman’s rank-order correlation coefficient was used to assess the relationships between the biomarkers of oxidative stress. The correlation between the concentration of ASMs or metabolites, expressed as Z-score, and the biomarkers of oxidative stress was assessed using Pearson’s correlation coefficient. Zi-scores were calculated using the equation $Z_{i}=\frac{\left(X_{i}-\bar{X}\right)}{SD}$, where x_i_ is the concentration of the ASM or its metabolite, $\bar{x}$ is the mean concentration, and SD is the standard deviation. The linear regression and Person’s correlation coefficient were calculated for each ASM. A *p*-value of less than 0.05 was considered statistically significant. IBM SPSS Statistics for Windows version 27 (IBM, Armonk, NY, USA) was used for statistical analysis.

## 3. Results

### 3.1. Demographic and Clinical Data

Demographic characteristics and clinical data of patients and controls are shown in Table 1. The study included 49 patients with epilepsy (29 men, 20 women) and 14 healthy controls (7 men, 7 women). The median age of the patients and control subjects was 40 and 40.5 years, respectively. The median duration of epilepsy was 18 years.

The patients were divided into a monotherapy and a polytherapy group, whereby the monotherapy group was additionally divided into groups according to treatment with the ASM generation. 43% of patients with epilepsy were treated with monotherapy and 57% with polytherapy. The first group included patients treated with the old generation of ASMs (n = 9), the second group included patients treated with the new generation of ASMs (n = 12), and finally, the third polytherapy group included patients treated with more than one ASM (n = 28). In the group of patients treated with the old generation ASMs, five patients were treated with CBZ and four patients with VPA. In the group of patients treated with the new generation of ASMs, eight patients were treated with LEV, two patients with PGB, and two patients with TPM. In the polytherapy group, twelve patients were treated with the combination of CBZ and one of the new ASMs (lacosamide, LTG, LEV, PGB, or TPM), six patients with the combination of LEV and one of the new ASMs (lacosamide, LTG, or OXC), six patients with the combination of VPA and one of the new ASMs (LEV or LTG), three patients with the combination of TPM and one of the new ASMs (LTG, OXC, or PGB), and one patient with the combination of CBZ, PHT, and TPM. The doses of CBZ, VPA, LEV, PGB, and TPM used in the treatment of the patients are listed in Table 1.

There was no statistically significant difference between patients and controls in terms of body weight (*p* = 0.888), body mass index (*p* = 0.597), age (*p* = 0.921), or gender (*p* = 0.577). In addition, there were no significant differences between patients on old ASMs, new ASMs, or polytherapy in terms of age (*p* = 0.613), gender (*p* = 0.917), body weight (*p* = 0.765), BMI (*p* = 0.538), or seizure frequency (*p* = 0.466). However, there were statistically significant differences between patients on old ASMs, new ASMs, and polytherapy in the duration of epilepsy (*p* = 0.024).

### 3.2. Biomarkers of Oxidative Stress in Patients Compared to Controls

The summarized data on oxidative stress parameters in patients and controls are shown in Table 2.

In patients, the enzyme activities of SOD and CAT were higher compared to controls (*p* < 0.001). On the other hand, the enzyme activities of GPx (*p* = 0.003) and GR (*p* < 0.001) were lower compared to controls.

Moreover, significantly increased levels of NO_3_^−^ + NO_2_^−^ and PC were observed compared to controls (*p* < 0.001). While NO_3_^−^ + NO_2_^−^ levels were increased, NO_2_^−^ levels were significantly lower in patients with epilepsy compared to controls (*p* = 0.005). On the other hand, MDA levels were not statistically significantly increased in the patient group compared to controls.

In addition, the observed GSH, GSSG, and GSH to GSSG ratios were significantly lower in the patients compared to the controls (*p* < 0.001, *p* = 0.014, and *p* = 0.032, respectively), as seen in Table 2.

SOD and CAT activities were positively correlated with MDA (r = 0.367, *p* = 0.014 and r = 0.391, *p* = 0.009, respectively), PC (r = 0.688, *p* = < 0.001 and r = 0.543, *p* < 0.001, respectively), and NO_3_^−^ + NO_2_^−^ (r = 0.622, *p* < 0.001 and r = 0.539, *p* < 0.001, respectively) levels, as shown in Table 3. In addition, a positive correlation was observed between SOD and CAT activities (r = 0.695, *p* < 0001), GPx and GR activities (r = 0.659, *p* < 0001), MDA and PC levels (r = 0.374, *p* = 0.012), and PC and NO_3_^−^ + NO_2_^−^ levels (r = 0.440, *p* < 0.002). On the other hand, SOD and CAT activities as well as MDA, PC, and NO_3_^−^ + NO_2_^−^ levels were negatively correlated with GPx (r = −0.822, *p* < 0.001, r = −0.739, *p* < 0.001, r = −0.360, *p* = 0.018, r = −0.711, *p* < 0.001 and r = −0.522, *p* < 0.001, respectively) and GR activities (r = −0.730, *p* < 0.001, r = −0.586, *p* < 0.001, r = −0.337, *p* = 0.025, r = −0.501, *p* < 0.001 and r = −0.431, *p* = 0.002, respectively).

### 3.3. Biomarkers of Oxidative Stress in Patients Treated with Different ASMs

Table 4 shows the comparison of oxidative stress parameters in patients treated with different generations of ASMs. The activity of SOD was the highest in the old generation of ASMs and the lowest in the new generation of ASMs. Statistical significance was observed between all compared groups (*p* < 0.001 in all comparisons). A similar response to ASM therapy was observed for CAT, but a significant difference was only found between the old and new generation ASMs and the old generation of ASMs and polytherapy (*p* < 0.007 in both comparisons). In contrast to SOD and CAT activities, GR and GPx activities were the lowest in the old group and the highest in the new ASM group. Statistical significance was observed between all compared groups (*p* < 0.01 in all comparisons).

In contrast to the enzymatic antioxidants, glutathione showed no response to the studied ASM therapy, neither in its reduced nor in its oxidized form.

The trend of higher oxidative damage to lipids was observed with the old generation ASM, although statistically insignificant. The MDA concentration was 13% higher in the old generation ASM group compared to the new generation (*p* = 0.061). However, oxidative damage to proteins was statistically different between all three ASM groups (*p* < 0.03 in all comparisons). We observed the highest PC concentrations in the old generation and the lowest in the new ASM group.

In addition, NO_3_^−^ + NO_2_^−^ concentrations were significantly different between the old and new generation ASMs and the old generation ASMs and polytherapy (*p* < 0.002 in both comparisons). We observed the highest concentrations in the old and comparable concentrations in the new generation ASMs and polytherapy groups. No differences were observed between the nitrite concentrations in the studied group of patients.

Plasma concentrations of CBZ, carbamazepine-10,11-epoxide, LEV, PGB, TPM, VPA, and 2-propyl-4-pentenoic acid were determined in patients with epilepsy treated with different ASMs. The mean plasma concentrations of CBZ (4.08 mg/L; range: 2.22–5.56 mg/L), carbamazepine-10,11-epoxide (0.71 mg/L; range: 0.22–1.41 mg/L), LEV (25.8 mg/L; range: 7.68–104 mg/L), PGB (2.80 mg/L; range: 1.58–3.95 mg/L), TPM (11.7 mg/L; range: 5.84–20.1 mg/L), VPA (76.7 mg/L; range: 24.0–140 mg/L), and 2-propyl-4-pentenoic acid (1.67 mg/L; range: 0.60–2.38 mg/L) were within the therapeutic range for CBZ (4–12 mg/L), LEV (12–46 mg/L), TPM (5–20 mg/L), and VPA (50–100 mg/L) [39]. The therapeutic range for PGB is not defined; however, the mean plasma concentrations of PGB determined in our study were clearly in the range (0.29–14.2 mg/L) determined in various published clinical studies [47,48].

**Figure 1:**
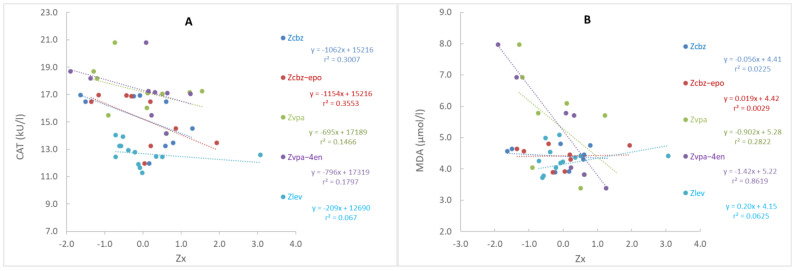
Correlation between catalase activity (**A**) or concertation of malondialdehyde (**B**) and concentrations of ASMs or their metabolites in patients with epilepsy.

The concentrations of ASMs or their metabolites, expressed as Z-scores, were associated with oxidative stress parameters (see Table S1). A trend of lower CAT activity with increasing carbamazepine-10,11-epoxide concentration was observed (*p* = 0.090). In addition, higher concentrations of the VPA metabolite 2-propyl-4-pentenoic acid were associated with a decrease in MDA concentrations (*p* = 0.003). The associations of all studied ASMs and metabolites with CAT activities or MDA concentrations are shown in Figure 1.

Zcbc, Zcbz-epo, Zvpa, Zvpa-4e, and Zlev are Z-scores for carbamazepine, carbamazepine-10,11-epoxide, valproic acid, 2-propyl-4-pentenoic acid, and levetiracetam concentrations, respectively. The linear regression equation and coefficients of determination (r^2^) are reported for each ASM and metabolite.

## 4. Discussion

Oxidative stress is a potential cytotoxic mechanism in epilepsy, particularly in neurodegenerative diseases, due to the vulnerability of neurons to reactive species [49]. It can contribute to epilepsy progression independently through mitochondrial dysfunction, leading to neuronal damage or death, which may propagate epileptic states [50,51,52]. Endogenous antioxidant systems are crucial in managing disease progression and treatment-related toxicities. SOD, CAT, GPx, and GR, along with glutathione (GSH), form the primary defense against reactive oxygen species. SOD converts superoxide radicals into hydrogen peroxide (H_2_O_2_), which CAT and GPx then neutralize. GPx oxidizes GSH to GSSG, which GR reduces back to GSH. Incomplete neutralization of H_2_O_2_ can cause oxidative damage to macromolecules that is detectable in vivo [53].

### 4.1. The Influence of ASMs Treatment on Oxidative Stress

ASMs are known to have various effects on the endogenous antioxidative system [54,55]. In addition, long-term use of some ASMs is thought to increase free radical formation and cause oxidative damage in neurons [18]. The impact of ASM treatment on oxidative stress has been extensively studied in patients with epilepsy compared to untreated patients or healthy controls [12,13,14,16,17,18,19,20,24,25,26,27,32,34,35,37,54,55,56,57]. However, the main limitation of most of these studies is that only old generation ASMs were included, in particular CBZ, PB, PHT, and VPA [12,13,14,18,19,24,25,27,32,34,35,54,55,56,57]. These ASMs have already been shown to induce oxidative stress either alone or via their metabolites [58]. Indeed, in in vitro models, these ASMs have been shown to produce reactive metabolites during metabolism that can covalently bind to various endogenous macromolecules or increase the formation of reactive oxygen species that can induce oxidative damage and cause toxicity [59,60]. This is particularly evident from the increased lipid peroxidation markers that were most noticeable with VPA treatment [16,25,27,34,54,55,57,61] and, to some extent, with CBZ [34,54,55], PB [7], and PHT [35,36]. In addition, Varoglu et al. reported increased 8-hydroxy-2′-deoxyguanosine levels in patients treated with CBZ and VPA compared to healthy controls [17]. Furthermore, there is also a lack of clinical studies in the literature investigating the effects of ASM polytherapy on oxidative stress levels in patients with epilepsy. Finally, it should be considered that approximately 30–40% of patients with epilepsy do not respond to monotherapy and therefore need to be treated with a combination of different ASMs, most commonly old and new generation ASMs [62].

In the proposed study, patients with epilepsy treated with CBZ, lacosamide, LEV, LTG, OXC, PGB, PHT, TMP, VPA, or their combinations showed an increased marker of biological macromolecule damage (PC), increased markers of NO metabolism (elevated NO_3_^−^ + NO_2_^−^, decreased NO_2_^−^), decreased glutathione concentration and ratio of reduced to oxidized glutathione form, and altered antioxidant enzyme activities in the entire patient group compared to healthy controls (Table 2).

The observed significant increase in SOD and CAT activities represents an enhanced endogenous enzymatic antioxidant response designed to increase endogenous free radical production. GPx is known to efficiently reduce H_2_O_2_ to water, but only when low, physiologically available amounts of H_2_O_2_ are present. When the amounts of H_2_O_2_ are higher, CAT plays a greater role in H_2_O_2_ removal [63,64]. This may explain the significant increase in CAT levels achieved. Since the main role in the degradation of H_2_O_2_ is assigned to CAT, it can be assumed that the GPx enzymatic antioxidant system plays a lesser role in the destruction of H_2_O_2_ and free radical scavenging. However, it can be expected that the activity of GPx will nevertheless increase [65]. Since GR is responsible for the reduction of GSSG and subsequent reactivation of oxidized GSH, an increase in GR activity is also expected. However, our results showed decreased GPx and GR activities, which could be due to increased protein inactivation or degradation [64]. The body’s antioxidant system is always striving to maintain the balance between superoxide radicals and H_2_O_2_ in cells, which is a result of normal metabolism [65]. Therefore, SOD and CAT activities, as well as GPx and GR activities, are expected to be linked for the reasons mentioned above. This was confirmed by the positive correlation observed between SOD and CAT on the one hand and GPx and GR on the other. Therefore, it can be assumed that the balance between SOD and CAT on the one hand and GPx on the other is more important than the absolute amount of the individual antioxidant enzymes [65].

Since the sum of NO_3_^−^ and NO_2_^−^ represents a marker for NO metabolism and thus the source of reactive nitrogen species (RNS), increased levels of NO_3_^−^ + NO_2_^−^ further confirm increased RNS and thus a possible cause for the development of oxidative stress. It has already been shown that prolonged exposure to elevated NO concentrations suppresses the mitochondrial respiratory chain and can also induce apoptosis and cell death [66]. On the other hand, the observed NO_2_^−^ levels were lower, suggesting increased consumption in patients with epilepsy compared to control subjects. NO_2_^−^ represents a biochemical NO reservoir that appears to be crucial for NO_2_^−^ derived cytoprotective effects, as confirmed by the abrogation of cytoprotective effects in animals pre-treated with NO scavengers [67]. Moreover, NO_2_^−^ has already been confirmed to have potent cytoprotective effects in ischemia and hypoxia, leading to cardiac, liver, brain, and kidney injury [68,69,70,71]. Therefore, NO appears to play a critical role in a number of physiological and pathophysiological processes in the brain, including modulation of neuronal plasticity, cerebral blood flow, cognitive and behavioral functions, and involvement in neurological disorders such as epilepsy [26].

Moreover, in our study, we observed a negative correlation of GPx and GR activity with SOD and CAT activity, as well as NO_3_^−^ + NO_2_^−^, MDA, and PC levels (Table 3), confirming the influence of oxidative stress on impaired action of the GPx and GR antioxidant defense systems, leading to an increase in markers of biological macromolecule oxidative damage. In addition, our study also confirmed a positive correlation between NO_3_^−^ + NO_2_^−^ concentrations and PC concentrations, as well as between PC and MDA concentrations, confirming the possible damaging effect of increased RNS on proteins and the joint increased oxidative damage to biological macromolecules. The observed positive correlation between the observed increase in SOD and CAT activities and MDA and PC concentrations indicates the ability of the endogenous enzyme antioxidant system to respond to increased oxidative stress. As mentioned above, SOD and CAT appear to be the main antioxidant enzymes responsible for maintaining the balance between the production of reactive species and the endogenous defense system in epilepsy. However, the negative correlation between the observed decrease in GPx and GR activities and the increase in MDA and PC concentrations indicates that the entire glutathione system is unable to combat the increased oxidative stress. This is also evident from the decrease in GSH concentrations in patients with epilepsy compared to control subjects. All these findings confirm the presence of increased oxidative stress in patients with epilepsy treated with ASMs (Table 2).

### 4.2. The Influence of Old and New Generation ASMs on Oxidative Stress

There are few studies in the literature investigating the influence of the new generation ASMs on oxidative stress markers in patients with epilepsy. They evaluate the effects of OXC [26,37], LTG [36], LEV [17,38], or TPM [33]. In addition, only a few studies have been published evaluating the impact of the combination of old and new generation ASMs, including CBZ, VPA, PB, PHT, clobazam, LTG, LEV, OXC, TPM, and VGB [11,16]. However, these studies do not provide results for individual ASM generations, but only for the entire group of patients using old and new generation ASMs. In this way, they do not allow a direct comparison and assessment of the potential differences between the two generations of ASMs in terms of oxidative stress [11,16]. In addition, some studies only evaluate one or at most two of the biomarkers of oxidative stress that we monitored [14,18,57,61,72], which is even more important in the case of a limited number of studies including the new generation of ASMs [17,33,36,38].

In our study, we observed increased levels of SOD and CAT and decreased levels of GPx and GR in patients treated with old or new generation ASMs (Table 4). Regarding markers of biological macromolecule damage, our study showed a significant increase in PC levels in patients treated with the old generation ASMs compared to those treated with the new generation ASMs. In addition, a trend towards elevated lipid damage was observed in patients treated with old generation ASMs versus new generation ASMs. Finally, our study showed a significant increase in NO_3_^−^ + NO_2_^−^ concentrations in patients treated with the old compared to the new generation ASMs.

The results of antioxidant enzyme activities, biological macromolecule damage marker concentrations, and NO_3_^−^ + NO_2_^−^ concentrations in the polytherapy group of patients with epilepsy are intermediate between those of patients treated with old generation ASMs and new generation ASMs (Table 4). The majority of patients in the polytherapy group were treated with a combination of both generations of ASMs. Based on the results of patients treated with only the old or the new generation, polytherapy is expected to result in a mixed effect of both generations. Moreover, epilepsy is known to have an impact on oxidative stress, and since there is a marked difference in the duration of epilepsy in the groups of patients treated with old and new generation ASMs (Table 1), it can be speculated that the observed differences in oxidative stress levels between these two groups could be a consequence of epileptic condition itself only. However, since the observed differences in oxidative stress markers (endogenous antioxidant enzyme activities of SOD, CAT, GPx, and GR, as well as markers of oxidative damage such as PC and NO metabolism—NO_3_^−^ + NO_2_^−^ concentration) between the polytherapy group and the group treated with old generation ASMs were confirmed, these results support the additional influence of ASM therapy on oxidative stress markers in addition to epilepsy itself. It is important to note that there were no statistically significant differences in the duration of epilepsy between the polytherapy group and the group treated with the old generation ASMs.

The novelty of the present study is also the association of oxidative stress parameters with concentrations of ASMs and their metabolites. We hypothesize that higher concentrations of carbamazepine-10,11-epoxide are associated with a decrease in CAT activity (Figure 1 and Table S1). Yip et al. showed that carbamazepine and carbamazepine-10,11-epoxide plasma concentrations were positively correlated with the formation of CBZ metabolite adducts on serum albumin in patients receiving CBZ therapy [73]. The proposed formation of protein adducts in patients on CBZ therapy indicates a possible mechanism of CAT inactivation. In addition, a negative correlation was found between VPA metabolite concentration and MDA plasma concentration, which is not the expected result since many studies have found increased MDA concentrations in patients on VPA therapy. However, in these studies, patients receiving VPA therapy were compared to healthy adults or patients receiving other ASM therapies, and since no concentrations of ASMs were measured, no correlation could be established between MDA concentration and the concentration of VPA or VPA metabolites.

It is important to consider that the observed inconsistency of the results obtained in our study with the results of published studies could be due to different analytical methods used to determine lipid peroxidation levels. For example, thiobarbituric acid reactive substances (TBARS) are a less reliable method than direct measurement of MDA due to the artificial formation of TBARS-like products under very “aggressive” conditions during the sample preparation procedure [74]. In addition, studies comparing oxidative stress parameters in patients with epilepsy largely differ in the population studied with respect to age [14,16,19,24,25,26,27,32,54,56] and type of epilepsy [22,32]. Menon et al. reported unchanged PC levels in patients with epilepsy treated with different ASMs compared to drug-naïve patients with epilepsy, demonstrating that ASMs have no effect on biological macromolecule markers of oxidative stress [11]. We hypothesize that this discrepancy with the results of our study may be mainly due to a large age difference in the population studied. The average age of our patients and control group was about twenty years older than in the study designed by Menon et al. Since oxidative damage increases with age physiologically and also due to some pathological processes, it is possible that there are large differences between comparable groups studied that differ in age. Menon et al. also showed no differences between NO concentrations in patients with epilepsy treated with different ASMs, untreated patients with epilepsy, and healthy controls [11]. This inconsistency with our study may be again due to age differences between the included patient and control populations compared to our studied population. Furthermore, Menon et al. only measured the nitrite content. We believe that total nitrate and nitrite levels serve as more meaningful parameters indicating total NO metabolism. Moreover, Peker et al. confirmed our results by observing increased NO_3_^−^ + NO_2_^−^ concentrations in patients treated with VPA compared to healthy controls [19].

The main limitations of this study were the unknown ASM history of patients prior to their inclusion in the study and the moderate sample size. We assume that three months of ASM treatment is sufficient to rule out the effects of previous ASM treatment on oxidative stress parameters. As this was an exploratory, i.e., hypothesis-generating study, our results can be used to design a larger study to further confirm the clinical relevance of the results obtained.

## 5. Conclusions

In conclusion, our study shows that significantly increased levels of SOD and CAT and significantly decreased levels of GR and GPx, as well as GSH concentrations, were observed in patients with epilepsy compared to control subjects. The observed increase in oxidative stress markers and the modulations of non-enzymatic antioxidant GSH concentrations and antioxidant enzyme activities in patients with epilepsy confirmed increased oxidative stress in patients with epilepsy compared to controls. Since the results of the proposed studies emphasize the impairment of the endogenous system and its inability to combat the increased oxidative stress in patients with epilepsy, it seems reasonable to evaluate the potential therapeutic effects of different approaches aimed at enhancing enzymatic activity or increasing the activity of the glutathione system. In addition, the results of our study showed a significantly greater increase in oxidative stress in patients treated with the old generation ASMs compared to patients treated with the new generation ASMs. It is already known that epilepsy itself can influence oxidative stress levels in patients with epilepsy. However, as the results of oxidative stress markers for the polytherapy group of patients in the proposed study are intermediate between the results of patients treated with the old and patients treated with the new generation ASMs, this confirms an important effect of ASMs on oxidative stress levels in patients with epilepsy.

Our study has shown that there is an urgent need to both evaluate the influence of the existing ASMs therapies on levels of oxidative stress in patients with epilepsy and to develop new therapies, including ASMs with potential adjunctive antioxidant and neuroprotective add-on effects.

## Figures and Tables

**Table 1:** Demographic characteristics of patients with epilepsy treated with different ASMs and controls.

	Patients with Epilepsy	Monotherapy		Poly-Therapy	Controls
		Old ASMs	New ASMs		
Subjects, n	49	9	12	28	14
^a^ Age [years], median (Q_1_–Q_3_)	40 (31–51)	42 (35–50.5)	43 (28–57.5)	37 (29.3–48.3)	40.5 (30–53.8)
^b^ Gender					
Male, n (%)	29 (59)	5 (56)	7 (58)	17 (61)	7 (50)
Female, n (%)	20 (41)	4 (44)	5 (42)	11 (39)	7 (50)
^a^ Body weight (kg), median (Q_1_–Q_3_)	68 (63–82)	75 (63–86)	64 (60.3–79.3)	69 (62.0–82.8)	72 (62.3–81.3)
^a^ BMI (kg/m^2^), median (Q_1_–Q_3_)	24.4 (21.9–26.6)	25.9 (22.2–28.5)	23.7 (20.0–25.9)	24.5 (21.8–26.4)	25.0 (21.9–27.1)
^a^ Duration of illness [years], median (Q_1_–Q_3_)	18 (10.5–24.5)	20 (17–32)	11 (7–18.3)	19 (14–26)	/
^a^ Seizure frequency, [seizure number/month], median (Q_1_–Q_3_)	0.7 (0–2)	0.1 (0–1)	0.4 (0–1.5)	2.5 (0–10)	/
ASM dose [mg/day]					
CBZ median, (Q_1_–Q_3_)	800 (400–1600)	800 (600–1600)	/	800 (800–1300)	/
VPA median, (Q_1_–Q_3_)	1250 (875–1625)	1000 (625–1375)	/	1500 (875–2250)	/
LEV median, (Q_1_–Q_3_)	1500 (1000–3000)	/	1000 (625–1000)	2000 (1500–3000)	/
PGB median, (Q_1_–Q_3_)	338 (300–600)	/	600 (/)	300 (300–356)	/
TPM median, (Q_1_–Q_3_)	225 (150–263)	/	175 (/)	250 (175–288)	/

ASMs—antiseizure medications; old ASMs—carbamazepine (CBZ), valproic acid (VPA); new ASMs—levetiracetam (LEV), pregabalin (PGB), topiramate (TPM). ^a^ Data presented median and Q_1_—first quartile (25th percentile); Q_3_—third quartile (75th percentile); ^b^ Categorical data reported as number, percentage of patients.

**Table 2:** Oxidative stress parameters in patients with epilepsy and control group.

Parameter	Patients with Epilepsy (n = 49)	Controls (n = 14)	*p* Value
SOD (U/mL)	202 (173–227)	142 (138–150)	<0.001
CAT (U/L)	14.1 (12.5–16.8)	11.7 (11.0–12.3)	<0.001
GR (U/L)	545 (452–595)	669 (651–702)	<0.001
GPx (U/L)	6003 (3925–9038)	8834 (7940–11,712)	0.003
GSH (µmol/L)	898 (574–1282)	1558 (1441–1826)	<0.001
GSSG (µmol/L)	199 (163–249)	264 (240–301)	0.014
GSH/GSSG	4.20 (3.33–5.96)	6.36 (5.39–7.07)	0.032
MDA (µmol/L)	4.39 (3.90–4.83)	4.35 (3.78–4.99)	0.757
PC (nmol/L)	42.5 (36.6–49.2)	21.0 (16.3–24.3)	<0.001
NO_3_^−^ + NO_2_^−^ (µmol/L)	54.3 (42.2–68.5)	23.3 (19.9–25.0)	<0.001
NO_2_^−^ (µmol/L)	1.96 (1.05–2.81)	3.08 (2.35–3.62)	0.005

CAT—catalase, GPx—Glutathione peroxidase, GR—Glutathione reductase, GSH—glutathione, GSH/GSSG—glutathione to glutathione disulfide ratio, GSSG—glutathione disulfide, MDA—malondialdehyde, NO_3_^−^ + NO_2_^−^—nitrate and nitrite, NO_2_^−^—nitrite, PC—protein carbonyl, SOD—superoxide dismutase. Data are presented as median and first (25th percentile) and third quartile (75th percentile) between the parenthesis.

**Table 3:** Bivariate rank-order correlations between various biomarkers of oxidative stress in patients with epilepsy treated with different ASMs. All values are Spearman′s rank-order coefficient, with *p*-value in parentheses.

	SOD	CAT	GR	GPx	GSH	GSSG	GSH/ GSSG	MDA	PC	NO_2_^−^ + NO_3_^−^	NO_2_
SOD	-										
CAT	0.695 (<0.001)	-									
GR	−0.730 (<0.001)	−0.586 (<0.001)	-								
GPx	−0.822 (<0.001)	−0.739 (<0.001)	0.659 (<0.001)	-							
GSH	ns	ns	ns	ns	-						
GSSG	ns	ns	ns	ns	0.376 (0.010)	-					
GSH/ GSSG	ns	ns	ns	ns	0.860 (<0.001)	ns	-				
MDA	0.367 (0.014)	0.391 (0.009)	−0.337 (0.025)	−0.360 (0.018)	ns	ns	ns	-			
PC	0.688 (<0.001)	0.543 (<0.001)	−0.501 (<0.001)	−0.711 (<0.001)	ns	ns	ns	0.374 (0.012)	-		
NO_2_^−^ + NO_3_^−^	0.622 (<0.001)	0.539 (<0.001)	−0.431 (0.002)	−0.522 (<0.001)	ns	ns	ns	ns	0.440 (0.002)	-	
NO_2_	ns	ns	ns	ns	ns	ns	ns	ns	ns	ns	-

CAT—catalase, GPx—Glutathione peroxidase, GR—Glutathione reductase, GSH—glutathione, GSH/GSSG—glutathione to glutathione disulfide ratio, GSSG—glutathione disulfide, MDA—malondialdehyde, NO_3_^−^ + NO_2_^−^—nitrate and nitrite, NO_2_^−^—nitrite, PC—protein carbonyl, SOD—superoxide dismutase. All values are Spearman′s rank-order correlation coefficient with *p*-value in parenthesis. ns—non-significant (*p* > 0.05).

**Table 4:** Oxidative stress parameters in patients treated with old ASMs, new generation of ASMs, or their combination (polytherapy).

Parameter	Old ASMs (n = 9)	New ASMs (n = 12)	Polytherapy (n = 28)	*p_G/G_*
SOD (U/mL)	292 (255–308)	173 (164–180)	206 (180–220)	O/N, O/P, N/P
CAT (U/L)	17.0 (16.5–18.4)	12.9 (11.4–14.0)	14.2 (12.4–16.9)	O/N, O/P
GR (U/L)	411 (388–469)	597 (583–655)	524 (452–591)	O/N, O/P, N/P
GPx (U/L)	2049 (1526–3414)	8976 (7697–12,396)	5572 (4161–8553)	O/N, O/P, N/P
GSH (µmol/L)	870 (790–1486)	961 (583–1320)	899 (566–1222)	ns
GSSG (µmol/L)	222 (182–244)	192 (150–237)	199 (159–268)	ns
GSH/GSSG	4.00 (3.30–8.00)	4.32 (3.59–6.25)	4.23 (3.01–5.96)	ns
MDA (µmol/L)	4.64 (4.17–6.35)	4.11 (3.67–4.41)	4.39 (3.95–5.01)	ns
PC (nmol/L)	53.8 (48.4–58.6)	34.4 (31.1–42.9)	39.0 (37.2–47.3)	O/N, O/P, N/P
NO_3_^−^ + NO_2_^−^ (µmol/L)	97.3 (73.6–106)	51.8 (40.6–55.5)	51.1 (42.1–64.0)	O/N, O/P
NO_2_^−^ (µmol/L)	1.66 (1.41–3.62)	1.96 (0.70–2.79)	2.02 (1.21–2.80)	ns

CAT—catalase, GPx—glutathione peroxidase, GR—glutathione reductase, GSH—glutathione, GSSG—glutathione disulfide, MDA—malondialdehyde, N—new ASMs, NO_3_^−^ + NO_2_^−^—nitrate and nitrite, NO_2_^−^—nitrite, ns—non-significant (*p* > 0.05), O—old ASMs, P—poly-therapy, PC—protein carbonyl, p_G/G_—significant difference between two generation of ASMs, SOD—superoxide dismutase. Data are presented as median and first (25th percentile) and third quartile (75th percentile) in the parenthesis.

## Data Availability

The authors agree to make the data and materials supporting the results or analyses presented in this paper available upon reasonable request.

## Supplementary Materials

The following supporting information can be downloaded at: https://www.mdpi.com/article/10.3390/medicina60081299/s1. Table S1: Bivariate rank-order correlations between various biomarkers of oxidative stress and Z-scores of ASM concentration in patients with epilepsy.

## References

[1] Fisher R.S., Acevedo C., Arzimanoglou A., Bogacz A., Cross J.H., Elger C.E., Engel J., Forsgren L., French J.A., Glynn M. (2014). ILAE Official Report: A practical clinical definition of epilepsy. Epilepsia.

[2] Scheffer I.E., Berkovic S., Capovilla G., Connolly M.B., French J., Guilhoto L., Hirsch E., Jain S., Mathern G.W., Moshé S.L. (2017). ILAE classification of the epilepsies: Position paper of the ILAE Commission for Classification and Terminology. Epilepsia.

[3] Yuksel A., Cengiz M., Seven M., Ulutin T. (2001). Changes in the antioxidant system in epileptic children receiving antiepileptic drugs: Two-year prospective studies. J. Child Neurol..

[4] Cárdenas-Rodríguez N., Coballase-Urrutia E., Rivera-Espinosa L., Romero-Toledo A., Sampieri A.I., Ortega-Cuellar D., Montesinos-Correa H., Floriano-Sánchez E., Carmona-Aparicio L. (2013). Modulation of antioxidant enzymatic activities by certain antiepileptic drugs (valproic acid, oxcarbazepine, and topiramate): Evidence in humans and experimental models. Oxid. Med. Cell Longev..

[5] Halliwell B. (1992). Reactive oxygen species and the central nervous system. J. Neurochem..

[6] Ashrafi M.R., Shams S., Nouri M., Mohseni M., Shabanian R., Yekaninejad M.S., Chegini N., Khodadad A., Safaralizadeh R. (2007). A probable causative factor for an old problem: Selenium and glutathione peroxidase appear to play important roles in epilepsy pathogenesis. Epilepsia.

[7] Aycicek A., Iscan A. (2007). The effects of carbamazepine, valproic acid and phenobarbital on the oxidative and antioxidative balance in epileptic children. Eur. Neurol..

[8] Ercegovac M., Jović N., Simić T., Beslać-Bumbaširević L., Sokić D., Savić-Radojević A., Matić M., Jovanović D., Ristić A., Đukić T. (2013). Antiepileptic drugs affect protein, lipid and DNA oxidative damage and antioxidant defense in patients with epilepsy. J. Med. Biochem..

[9] Güneş S., Dirik E., Yiş U., Seçkin E., Kuralay F., Köse S., Ünalp A. (2009). Oxidant status in children after febrile seizures. Pediatr. Neurol..

[10] Mehmet U.C., Sefer V., Yavuz Y., Esref A., Tahsin C., Adalet A., Hatice Y., Mehmet U.A. (2012). Serum paroxonase-1 activities and malondialdehyde levels in patients with epilepsy. Dicel Med. J..

[11] Menon B., Ramalingam K., Kumar R.V. (2012). Oxidative stress in patients with epilepsy is independent of antiepileptic drugs. Seizure.

[12] Pandey K.M., Mittra P., Maheshwari P.K. (2012). The Lipid Peroxidation Product as a Marker of Oxidative Stress in Epilepsy. J. Clin. Diagn. Res..

[13] Sudha K., Rao A.V., Rao A. (2001). Oxidative stress and antioxidants in epilepsy. Clin. Chim. Acta.

[14] Verrotti A., Scardapane A., Franzoni E., Manco R., Chiarelli F. (2008). Increased oxidative stress in epileptic children treated with valproic acid. Epilepsy Res..

[15] Kösem A., Yücel C., Titiz A.P., Sezer S., Neşelioğlu S., Erel Ö., Turhan T. (2021). Evaluation of serum thiol-disulphide homeostasis parameters as oxidative stress markers in epilepsy patients. Acta Neurol. Belg..

[16] Turkdogan D., Toplan S., Karakoc Y. (2002). Lipid peroxidation and antioxidative enzyme activities in childhood epilepsy. J. Child Neurol..

[17] Varoglu A.O., Yildirim A., Aygul R., Gundogdu O.L., Sahin Y.N. (2010). Effects of valproate, carbamazepine, and levetiracetam on the antioxidant and oxidant systems in epileptic patients and their clinical importance. Clin. Neuropharmacol..

[18] Schulpis K.H., Lazaropoulou C., Regoutas S., Karikas G.A., Margeli A., Tsakiris S., Papassotiriou I. (2006). Valproic acid monotherapy induces DNA oxidative damage. Toxicology.

[19] Peker E., Oktar S., Arı M., Kozan R., Doğan M., Çağan E., Söğüt S. (2009). Nitric oxide, lipid peroxidation, and antioxidant enzyme levels in epileptic children using valproic acid. Brain Res..

[20] Menon B., Ramalingam K., Kumar R.V. (2014). Low plasma antioxidant status in patients with epilepsy and the role of antiepileptic drugs on oxidative stress. Ann. Indian. Acad. Neurol..

[21] Ercegovac M., Jovic N., Simic T., Beslac-Bumbasirevic L., Sokic D., Djukic T., Savic-Radojevic A., Matic M., Mimic-Oka J., Pljesa-Ercegovac M. (2010). Byproducts of protein, lipid and DNA oxidative damage and antioxidant enzyme activities in seizure. Seizure.

[22] Lopez J., Gonzalez M.E., Lorigados L., Morales L., Riveron G., Bauza J.Y. (2007). Oxidative stress markers in surgically treated patients with refractory epilepsy. Clin. Biochem..

[23] Nemade S.T., Melinkeri R.R. (2010). Oxidative and Antioxidative Status in Epilepsy. Prevara Med. Rev..

[24] Verrotti A., Basciani F., Trotta D., Pomilio M.P., Morgese G., Chiarelli F. (2002). Serum copper, zinc, selenium, glutathione peroxidase and superoxide dismutase levels in epileptic children before and after 1 year of sodium valproate and carbamazepine therapy. Epilepsy Res..

[25] Zhang Y.-J., Zhang M., Wang X.-C., Yu Y.-H., Jin P.-J., Wang Y. (2011). Effects of sodium valproate on neutrophils’ oxidative metabolism and oxidant status in children with idiopathic epilepsy. Zhonghua Er Ke Za Zhi.

[26] Arhan E., Serdaroglu A., Ozturk B., Ozturk H.S., Ozcelik A., Kurt N., Kutsal E., Sevinc N. (2011). Effects of epilepsy and antiepileptic drugs on nitric oxide, lipid peroxidation and xanthine oxidase system in children with idiopathic epilepsy. Seizure.

[27] Yis U., Seckin E., Kurul S.H., Kuralay F., Dirik E. (2009). Effects of epilepsy and valproic acid on oxidant status in children with idiopathic epilepsy. Epilepsy Res..

[28] Martinc B., Grabnar I., Vovk T. (2015). Antioxidants as a Preventive Treatment for Epileptic Process: A Review of the Current Status. Curr. Neuropharmacol..

[29] Miziak B., Blaszczyk B., Chroscinska-Krawczyk M., Danilkiewicz G., Jagiello-Wojtowicz E., Czuczwar S.J. (2014). The problem of osteoporosis in epileptic patients taking antiepileptic drugs. Expert. Opin. Drug Saf..

[30] Eddy C.M., Rickards H.E., Cavanna A.E. (2011). The cognitive impact of antiepileptic drugs. Ther. Adv. Neurol. Disord..

[31] Jakovljević D., Nikolić M., Jovanović V., Uzelac T.V., Nikolić-Kokić A., Novaković E., Miljević Ć., Milovanović M., Blagojević D. (2024). Influence of Long-Term Anti-Seizure Medications on Redox Parameters in Human Blood. Pharmaceuticals.

[32] Sobaniec W., Solowiej E., Kulak W., Bockowski L., Smigielska-Kuzia J., Artemowicz B. (2006). Evaluation of the influence of antiepileptic therapy on antioxidant enzyme activity and lipid peroxidation in erythrocytes of children with epilepsy. J. Child Neurol..

[33] Yurekli V.A., Naziroglu M. (2013). Selenium and topiramate attenuates blood oxidative toxicity in patients with epilepsy: A clinical pilot study. Biol. Trace Elem. Res..

[34] Yuksel A., Cengiz M., Seven M., Ulutin T. (2000). Erythrocyte glutathione, glutathione peroxidase, superoxide dismutase and serum lipid peroxidation in epileptic children with valproate and carbamazepine monotherapy. J. Basic. Clin. Physiol. Pharmacol..

[35] Liu C.S., Wu H.M., Kao S.H., Wei Y.H. (1998). Serum trace elements, glutathione, copper/zinc superoxide dismutase, and lipid peroxidation in epileptic patients with phenytoin or carbamazepine monotherapy. Clin. Neuropharmacol..

[36] Lu W., Uetrecht J.P. (2007). Possible bioactivation pathways of lamotrigine. Drug Metab. Dispos..

[37] Bolayir E., Celik K., Tas A., Topaktas S., Bakir S. (2004). The effects of oxcarbazepine on oxidative stress in epileptic patients. Methods Find. Exp. Clin. Pharmacol..

[38] Ozden H., Kabay S.C., Toker A., Ustüner M.C., Ozbayer C., Ustüner D., Günes H.V. (2010). The effects of levetiracetam on urinary 15f-2t-isoprostane levels in epileptic patients. Seizure.

[39] Patsalos P.N., Berry D.J., Bourgeois B.F.D., Cloyd J.C., Glauser T.A., Johannessen S.I., Leppik I.E., Tomson T., Perucca E. (2008). Antiepileptic drugs—Best practice guidelines for therapeutic drug monitoring: A position paper by the subcommission on therapeutic drug monitoring, ILAE Commission on Therapeutic Strategies. Epilepsia.

[40] Czauderna M., Kowalczyk J., Marounek M. (2011). The simple and sensitive measurement of malondialdehyde in selected specimens of biological origin and some feed by reversed phase high performance liquid chromatography. J. Chromatogr. B Analyt Technol. Biomed. Life Sci..

[41] Squellerio I., Caruso D., Porro B., Veglia F., Tremoli E., Cavalca V. (2012). Direct glutathione quantification in human blood by LC-MS/MS: Comparison with HPLC with electrochemical detection. J. Pharm. Biomed. Anal..

[42] Aebi H. (1984). Catalase in vitro. Methods Enzymol..

[43] Leite C.E., Petersen G.O., Lunardelli A., Thiesen F.V. (2009). A high-performance liquid chromatography method for the determination of carbamazepine and carbamazepine-10,11-epoxide and its comparison with chemiluminescent immunoassay. Clin. Chem. Lab. Med..

[44] Contin M., Mohamed S., Albani F., Riva R., Baruzzi A. (2008). Simple and validated HPLC-UV analysis of levetiracetam in deproteinized plasma of patients with epilepsy. J. Chromatogr. B Analyt Technol. Biomed. Life Sci..

[45] Martinc B., Roškar R., Grabnar I., Vovk T. (2014). Simultaneous determination of gabapentin, pregabalin, vigabatrin, and topiramate in plasma by HPLC with fluorescence detection. J. Chromatogr. B Analyt Technol. Biomed. Life Sci..

[46] Sasamot K., Ushijima T., Saito M., Ohkura Y. (1996). Precolumn Fluorescence Derivatization of Carboxylic Acids Using 4-Aminomethyl-6,7-dimethoxycoumarin in a Two-Phase Medium. Anal. Sci..

[47] Arroyo S., Anhut H., Kugler A.R., Lee C.M., Knapp L.E., Garofalo E.A., Messmer S. (2004). Pregabalin Add-on Treatment: A Randomized, Double-blind, Placebo-controlled, Dose-Response Study in Adults with Partial Seizures. Epilepsia.

[48] Berry D., Millington C. (2005). Analysis of pregabalin at therapeutic concentrations in human plasma/serum by reversed-phase HPLC. Ther. Drug Monit..

[49] Martinc B., Grabnar I., Vovk T. (2012). The Role of Reactive Species in Epileptogenesis and Influence of Antiepileptic Drug Therapy on Oxidative Stress. Curr. Neuropharmacol..

[50] Jarrett S.G., Liang L.P., Hellier J.L., Staley K.J., Patel M. (2008). Mitochondrial DNA damage and impaired base excision repair during epileptogenesis. Neurobiol. Dis..

[51] Patel M.N. (2002). Oxidative stress, mitochondrial dysfunction, and epilepsy. Free Radic. Res..

[52] Rowley S., Patel M. (2013). Mitochondrial involvement and oxidative stress in temporal lobe epilepsy. Free Radic. Biol. Med..

[53] Baram T.Z. (2012). The brain, seizures and epilepsy throughout life: Understanding a moving target. Epilepsy Curr..

[54] Hamed S.A., Abdellah M.M., El-Melegy N. (2004). Blood levels of trace elements, electrolytes, and oxidative stress/antioxidant systems in epileptic patients. J. Pharmacol. Sci..

[55] Solowiej E., Sobaniec W. (2003). The effect of antiepileptic drug therapy on antioxidant enzyme activity and serum lipid peroxidation in young patients with epilepsy. Neurol. Neurochir. Pol..

[56] Cengiz M., Yuksel A., Seven M. (2000). The effects of carbamazepine and valproic acid on the erythrocyte glutathione, glutathione peroxidase, superoxide dismutase and serum lipid peroxidation in epileptic children. Pharmacol. Res..

[57] Martínez-Ballesteros C., Pita-Calandre E., Sánchez-González Y., Rodríguez-López C.M., Agil A. (2004). Lipid peroxidation in adult epileptic patients treated with valproic acid. Rev. Neurol..

[58] Santos N.A., Medina W.S., Martins N.M., Rodrigues M.A., Curti C., Santos A.C. (2008). Involvement of oxidative stress in the hepatotoxicity induced by aromatic antiepileptic drugs. Toxicol. Vitr..

[59] Bjornsson E. (2008). Hepatotoxicity associated with antiepileptic drugs. Acta Neurol. Scand..

[60] Lu W., Uetrecht J.P. (2008). Peroxidase-mediated bioactivation of hydroxylated metabolites of carbamazepine and phenytoin. Drug Metab. Dispos..

[61] Michoulas A., Tong V., Teng X.W., Chang T.K., Abbott F.S., Farrell K. (2006). Oxidative stress in children receiving valproic acid. J. Pediatr..

[62] Kwan P., Brodie M.J. (2006). Combination therapy in epilepsy: When and what to use. Drugs.

[63] Halliwell B., Gutteridge J.M. (2008). Reactive species can pose special problems needing special solutions: Some examples. Free Radicals in Biology and Medicine.

[64] Rumià J., Marmol F., Sanchez J., Giménez-Crouseilles J., Carreño M., Bargalló N., Boget T., Pintor L., Setoain X., Donaire A. (2013). Oxidative stress markers in the neocortex of drug-resistant epilepsy patients submitted to epilepsy surgery. Epilepsy Res..

[65] Erakovic V., Zupan G., Varljen J., Laginja J., Simonic A. (2000). Lithium plus pilocarpine induced status epilepticus—Biochemical changes. Neurosci. Res..

[66] Walford G.A., Moussignac R.L., Scribner A.W., Loscalzo J., Leopold J.A. (2004). Hypoxia potentiates nitric oxide-mediated apoptosis in endothelial cells via peroxynitrite-induced activation of mitochondria-dependent and -independent pathways. J. Biol. Chem..

[67] Jones S.P., Bolli R. (2006). The ubiquitous role of nitric oxide in cardioprotection. J. Mol. Cell Cardiol..

[68] Dezfulian C., Raat N., Shiva S., Gladwin M.T. (2007). Role of the anion nitrite in ischemia-reperfusion cytoprotection and therapeutics. Cardiovasc. Res..

[69] Lundberg J.O., Weitzberg E., Gladwin M.T. (2008). The nitrate-nitrite-nitric oxide pathway in physiology and therapeutics. Nat. Rev. Drug Discov..

[70] van Faassen E.E., Bahrami S., Feelisch M., Hogg N., Kelm M., Kim-Shapiro D.B., Kozlov A.V., Li H., Lundberg J.O., Mason R. (2009). Nitrite as regulator of hypoxic signaling in mammalian physiology. Med. Res. Rev..

[71] Webb A., Bond R., McLean P., Uppal R., Benjamin N., Ahluwalia A. (2004). Reduction of nitrite to nitric oxide during ischemia protects against myocardial ischemia-reperfusion damage. Proc. Natl. Acad. Sci. USA.

[72] Ono H., Sakamoto A., Sakura N. (2000). Plasma total glutathione concentrations in epileptic patients taking anticonvulsants. Clin. Chim. Acta.

[73] Yip V.L.M., Meng X., Maggs J.L., Jenkins R.E., Marlot P.T., Marson A.G., Park B.K., Pirmohamed M. (2017). Mass Spectrometric Characterization of Circulating Covalent Protein Adducts Derived from Epoxide Metabolites of Carbamazepine in Patients. Chem. Res. Toxicol..

[74] Liu J., Yeo H.C., Doniger S.J., Ames B.N. (1997). Assay of aldehydes from lipid peroxidation: Gas chromatography-mass spectrometry compared to thiobarbituric acid. Anal. Biochem..

