# Supplementary material for: A Cross-Sectional Study Comparing Oxidative Stress in Patients with Epilepsy Treated with Old and New Generation Antiseizure Medications
Source: Medicina (Kaunas). 2024 Aug 12;60(8):1299. doi: 10.3390/medicina60081299 (PMC11356379; doi:10.3390/medicina60081299)
Supplement: Supplementary file 1 [file medicina-60-01299-s001.zip › medicina-3110064-supplementary.pdf]

Supplementary, Table S1. Bivariate rank-order correlations between various biomarkers of oxidative stress and Z-scores of ASM concentration in patients with epilepsy.

	**Zcbz** (n=9)	**Zcbz-epo** (n=9)	**Zvpa** (n=10)	**Zvpa-4en** (n=9)	**Zlev** (n=13)
**Antioxidant enzymes**					
**SOD**	-0.339 (0.373)	-0.359 (0.343)	-0.280 (0.433)	-0.351 (0.354)	0.298 (0.322)
**CAT**	-0.548 (0.126)	-0.596 (0.090)	-0.383 (0.275)	-0.424 (0.255)	-0.259 (0.393)
**GR**	0.222 (0.567)	0.349 (0.357)	0.411 (0.238)	0.316 (0.407)	-0.232 (0.445)
**GPx**	0.293 (0.445)	0.212 (0.583)	-0.047 (0.897)	0.122 (0.755)	0.280 (0.355)
**Glutathione system**					
**GSH**	0.148 (0.727)	0.062 (0.885)	0.188 (0.603)	0.037 (0.925)	-0.195 (0.532)
**GSSG**	0.098 (0.802)	0.209 (0.589)	-0.300 (0.399)	-0.057 (0.884)	-0.331 (0.270)
**GSH/ GSSG**	0.277 (0.506)	0.040 (0.925)	0.325 (0.359)	0.194 (0.618)	-0.038 (0.901)
**Oxidative damage**					
**MDA**	-0.150 (0.723)	0.054 (0.900)	-0.531 (0.175)	**-0.928 (0.003)**	0.250 (0.410)
**PC**	-0.482 (0.189)	-0.549 (0.126)	0.357 (0.312)	-0.127 (0.754)	-0.244 (0.422)
**Nitric oxide**					
**NO_2^- + NO_3^-**	-0.218 (0.537)	-0.323 (0.396)	-0.280 (0.434)	-0.238 (0.537)	0.274 (0.365)
**NO_2**	-0.504 (0.167)	-0.285 (0.458)	-0.218 (0.545)	0.161 (0.679)	-0.081 (0.794)

CAT – catalase, GPx – Glutathione peroxidase, GR – Glutathione reductase, GSH – glutathione, GSH/GSSG – glutathione to glutathione disulfide ratio, GSSG – glutathione disulfide, MDA – malondialdehyde, NO_3^- + NO_2^- - nitrate and nitrite, NO_2^- - nitrite, PC – protein carbonyl, SOD – superoxide dismutase.
All values are Pearson's correlation coefficient with *p*-value in parentheses.
